# European Collaborative and Interprofessional Capability Framework for Prevention and Management of Frailty—a consensus process supported by the Joint Action for Frailty Prevention (ADVANTAGE) and the European Geriatric Medicine Society (EuGMS)

**DOI:** 10.1007/s40520-019-01455-5

**Published:** 2020-01-22

**Authors:** Regina Roller-Wirnsberger, Sonja Lindner, Aaron Liew, Ronan O’Caoimh, Maria-Lamprini Koula, Dawn Moody, Juan Manuel Espinosa, Thérèse van Durme, Plamen Dimitrov, Tomislav Benjak, Elena Nicolaidou, Teija Hammar, Eliane Vanhecke, Ulrike Junius-Walker, Péter Csizmadia, Lucia Galluzzo, Jūratė Macijauskienė, Mohamed Salem, Liset Rietman, Anette Hylen Ranhoff, Tomasz Targowski, Miguel Telo de Arriaga, Elena Bozdog, Branko Gabrovec, Anne Hendry, Finbarr C. Martin, Leocadio Rodriguez-Mañas

**Affiliations:** 1 Department of Internal Medicine, Medical University of Graz, Auenbruggerplatz 15, 8036 Graz, Austria; 2 Health Service Executive of Ireland, Dublin, Ireland; 3 National University of Ireland, Galway, Ireland; 4 Company of Psychosocial Research and Intervention, Ioannina, Greece; 5 National Health Services Orkney, Orkney, Scotland; 6 Regional Ministry of Health of Andalusia, Sevilla, Spain; 7 Catholic University of Louvain, Institute of Health and Society, Brussels, Belgium; 8 National Center of Public Health and Analyses, Sofia, Bulgaria; 9 Croatian Institute of Public Health, Zagreb, Croatia; 10 Ministry of Health of the Republic of Cyprus, Nicosia, Cyprus; 11 Finnish Institute for Health and Welfare, Helsinki, Finland; 12 Ministry of Health and Social Solidarity, General Directorate for Health, Paris, France; 13 Medical University of Hannover, Hannover, Germany; 14 Ministry of Human Capacities, Budapest, Hungary; 15 Italian National Health Institute, Rome, Italy; 16 Lithuanian University of Health Sciences, Kaunas, Lithuania; 17 San Vincent De Paule Long Term Care Facility, Marsa, Malta; 18 National Institute for Public Health and the Environment, Bilthoven, The Netherlands; 19 Norwegian Institute of Public Health, Oslo, Norway; 20 National Institute of Geriatrics, Rheumatology and Rehabilitation, Warsaw, Poland; 21 Ministry of Health, Directorate-General of Health, Lisbon, Portugal; 22 Babeș-Bolyai University, Cluj-Napoca, Romania; 23 National Institute of Public Health, Ljubljana, Slovenia; 24 National Health Service Lanarkshire, Scotland, UK; 25 European Geriatric Medicine Society (EuGMS), Genoa, Italy; 26 Hospital Universitario de Getafe, Getafe, Spain

**Keywords:** Education, Training, Competences, Multi-professional, Frailty management, Frailty prevention

## Abstract

**Background:**

Interprofessional collaborative practice (ICP) is currently recommended for the delivery of high-quality integrated care for older people. Frailty prevention and management are key elements to be tackled on a multi-professional level.

**Aim:**

This study aims to develop a consensus-based European multi-professional capability framework for frailty prevention and management.

**Methods:**

Using a modified Delphi technique, a consensus-based framework of knowledge, skills and attitudes for all professions involved in the care pathway of older people was developed within two consultation rounds. The template for the process was derived from competency frameworks collected in a comprehensive approach from EU-funded projects of the European Commission (EC) supported best practice models for health workforce development.

**Results:**

The agreed framework consists of 25 items structured in 4 domains of capabilities. Content covers the understanding about frailty, skills for screening and assessment as well as management procedures for every profession involved. The majority of items focused on interprofessional collaboration, communication and person-centred care planning.

**Discussion:**

This framework facilitates clarification of professionals’ roles and standardizes procedures for cross-sectional care processes. Despite a lack of evidence for educational interventions, health workforce development remains an important aspect of quality assurance in health care systems.

**Conclusions:**

The multi-professional capability framework for frailty prevention and management incorporated interprofessional collaborative practice, consistent with current recommendations by the World Health Organization, Science Advice for Policy by European Academies and the European Commission.

## Background

People at similar chronological age often present with heterogenous biological ageing phenotypes, due to various factors including different life courses, functional abilities and comorbidity. Consequently, frailty has gained increasing interest among health and social care professionals, scientists, public health experts and care planners, highlighting the diversity in self-care capacity among older adults [1].

In fact, the European Commission (EC) has prioritized frailty within the health policy agenda of the majority of the European Union (EU) member states through its “Joint Action on Frailty Prevention” (ADVANTAGE JA) consortium [2]. Despite the absence of evidence supporting education and training programs for professionals involved within the care pathway for older people [3], the consortium assumed that a common competency framework for different professions would support interprofessional collaborative practice (ICP) for integrated and high-quality care for older people [4]. ICP differs from inter- or multi-professional collaboration in terms of hierarchy, role clarification of team members, responsibilities within the team, communication structures and interactions with patients and relatives. Hierarchy structures in ICP teams are flat with no central leader and clear role description for members. ICP is relationship focused and community based. Based on this concept, a set of general and interprofessional core competencies has been discussed for all professions involved in the health care pathways [4].

Furthermore, Ellis and Sevdalis also recommended the development of frameworks for the management of older people to create a capacity to build strong multi-professional teams for the care of older people in different settings [5].

The current study describes the development of a collaborative and multi-professional capability framework for prevention and management of frailty. The study was developed under the auspices of the ADVANTAGE JA in collaboration with the European Geriatric Medicine Society (EuGMS) and aims at establishing multi-professional training standards for improving quality of care of older people.

## Methods

The development of the European Collaborative Interprofessional Capability Framework for Frailty Prevention and Management is based on a consensus process via a modified Delphi technique [6]. A core group of six experts was nominated by the ADVANTAGE JA and the European Geriatric Medicine Society (EuGMS) to guide the process. The group was responsible for the literature survey to develop the template, the conduct of the consensus process, the evaluation of intermediate feedbacks from participants, the communication within the consensus participant panel as well as the summary of results in this publication. This core group consisted of two experts from Austria (RR-W, SL), two experts from Ireland (AL, RC), one expert from United Kingdom (DM) and one expert from Greece (MK).

### Selection of experts to run the Delphi survey

Expert choice for creating a consensus was justified upon their involvement in the ADVANTAGE JA and their expertise in the field of frailty management and prevention. Furthermore, members of the Special Interest Group (SIG) in Education and Training of EuGMS were evaluated for their participation in the process. In total, 25 experts from 22 European countries were involved in this process.

### Development of the template

The items used in the template for the Delphi process were derived from programs which had been identified as best practice models for frailty prevention in Europe by the ADVANTAGE JA [7]: Capability Framework for Frailty Prevention–UK [8], Sunfrail Project [9], Frailty 360 ° Project [10], FACET Project [11], Frailty training events–UK [12], National Frailty Education Programme–Ireland [13], Frailty for Healthcare Professionals–UK [14], Frailty Training Programs–France [15], Education Module Frailty–UK [16], Postgraduate Certificate in Acute Care of the Older Person with Frailty–UK [17], MSc Specialist Practice Frail older Adults for Health and Social Care–UK [18], Training Programme for Health Care Professionals on detecting pre-frailty and recognizing the initial steps of frailty on primary care–Spain [19], +AGIL–Spain [20], Medical Science Frailty and Integrated Care–UK [21], Perssilaa Project [22] and Frailty Risk Screening in community dwelling older People–France [23]. The single capabilities outlined in the given curricula and catalogues were listed and merged by the core group (data not shown). This exhaustive approach enabled the inclusion and consideration of multiple professions for successful frailty prevention and management, such as geriatricians, physicians, psychologists, public health specialists, dieticians and nursing scientists. Table 1 shows the template for the first round of the Delphi Survey.

**Table 1:** Template for the first round of the Delphi Survey

Item	Inclusion?	Comment
*Frailty core capabilities framework*		
1. Understand frailty		
1.1 Definition and prevalence		
1.1.1. Knowing that as a construct, frailty is an age-associated condition of reduced resilience and increased vulnerability to adverse events	Please choose an element	
1.1.2. Knowing that frailty becomes more frequent with ageing and can be defined through the frailty phenotype and the “cumulative deficit” models of frailty	Please choose an element	
1.2 Disability, multimorbidity and dependency		
1.2.1. Understand the concept of frailty as a multidimensional condition and recognize its individual nature and stages including all determinants of health identified by the WHO	Please choose an element	
1.2.2. Understand that as a construct, frailty is potentially reversible with recognized transitional stages from robust, and pre-frail through to end of life	Please choose an element	
1.2.3. Knowing that the trajectories of frailty are influenced by lifestyle and other factors including the risk of frailty syndromes such as confusion, falls, incontinence, problems with mobility and side effects of medication	Please choose an element	
1.3 Personal impact		
1.3.1. Understanding the multidimensional, heterogeneous nature of frailty and its bidirectional relationship with many different aspects of a person’s life (including multimorbidity, functional ability, physical health, psychosocial health and cognitive function)	Please choose an element	
2. Identification of frailty		
2.1. Screening, diagnosing and assessment		
2.1.1. Apply common tools suggested in the Frailty Prevention Approach (FPA) document to support the identification and the process of assessment (CGA) of frailty severity including as part of an integrated care approach	Please choose an element	
2.1.2. Knowing that frailty assessment should include consideration of the potential use of assistive technology (AT)	Please choose an element	
2.1.3. Understand that frailty syndromes may be a first presentation or first sign of frailty	Please choose an element	
2.1.4. Understand the importance of early recognition and timely management of frailty syndromes	Please choose an element	
3. Person-centred collaborative working		
3.1. Person-centred approaches including communication		
3.1.1. Understand that person-centred care includes all elements of a person’s life that are important to them	Please choose an element	
3.1.2. Understand the implications of relevant legislation and guidance for consent and shared decision-making (e.g. mental capacity legislation)	Please choose an element	
3.1.3. Person-centred care requires being able to communicate verbally and on a non-verbal basis with older people to achieve shared decision-making in the FPA	Please choose an element	
3.1.4. Demonstrate effective communication with family and carers to support them in their individual care-giving role	Please choose an element	
3.2. Collaborative and integrated working		
3.2.1. Be able to work in partnership with others, exploring and integrating the views across multidisciplinary teams and organizations to deliver care in a coordinated and integrated way, showing an understanding of the role of others	Please choose an element	
3.2.2. Be able to share information with other professionals, including an older person’s wishes, in a timely and appropriate manner, considering issues of consent and confidentiality	Please choose an element	
4. Managing frailty and its prevention		
4.1. Preventing and reducing the risk of frailty progression		
4.1.1. Know interventions to improve independence and quality of life for people at risk or living with frailty, including social and economic factors, exercise, physical activity, diet, hydration and proper drug management for preventing and reducing the progression of frailty.	Please choose an element	
4.1.2. Be able to measure, monitor and report important measures of frailty outcomes in different settings including all determinants of health	Please choose an element	
4.2. Living well		
4.2.1. Understand the concept and principles of a community development, asset-based approach to care and support for older people at risk of frailty or those already living with frailty	Please choose an element	
4.3. Promoting independence		
4.3.1. Be able to provide specific advice and guidance on changing or adapting the physical and social environment to ensure physical safety, comfort and emotional security	Please choose an element	
4.4. Community skills		
4.4.1. Be able to promote the benefits of developing community skills and engaging with the local community, amongst colleagues and senior managers/board members in relation to improving outcomes for people living with frailty and those important to them	Please choose an element	
4.5. Care and support planning		
4.5.1. Understand the importance of care and support planning being a “holistic” and person-centred process at all levels of care that needs to be reviewed regularly	Please choose an element	
4.6. Research and evidence-based practice		
4.6.1. Understand the reasons for conducting service evaluation and research and be able to participate in service evaluation and research in the workplace	Please choose an element	
4.6.2. Understand how local and national policy and the outcomes of research in frailty care and support can inform and impact on workplace practices and care delivery	Please choose an element	
4.7. Leadership in transforming services		
4.7.1. Understand the importance of continuing professional development to ensure the methods used for preventing frailty are robust, valid and reliable	Please choose an element	
4.7.2. Understand that everyone has a part to play in supporting people living with frailty to have the best possible quality of life	Please choose an element	
4.7.3. Be able to provide support for colleagues to develop their skills and confidence when working with older people at possible risk of frailty and those important to them	Please choose an element	
4.7.4. Be able to use people’s feedback and person-centred outcomes to coproduce investments in services with those who use them	Please choose an element	
4.7.5. Recognize the importance of effective clinical governance which involves all stakeholders for overall management of frailty	Please choose an element	

This template included four domains based upon professional competences from projects and best practice models and represents a comprehensive overview of current content in literature for professional competences in frailty prevention and management

### Delphi process

The core group pre-defined a consensus rate of > 70% for the integration of a competency into the framework. In this Delphi survey, participants were instructed to rate “yes” or “no” for each item, denoting the inclusion into or exclusion from the framework and to comment on the current wording or suggest new items (Table 1). Free comments were considered, if the item concerned was rated “yes” and/or a reference to the current wording of the item was given by more than 10% of the participants [6]. The participants in the consensus process were granted a deadline of 2 weeks for replying and possibly discussing the items within their institutional teams during each Delphi round.

#### First Delphi round

Following an implied consent via a reply from an email invitation, the first Delphi round was conducted in February 2019 using the paper-based questionnaire as shown in Table 1. The survey included 6 items on general considerations in the first domain, 4 items on identification of frailty-associated signs and symptoms in the second domain, 6 items on multi-professional collaboration in the third domain and 13 items on management and leadership skills in the fourth domain.

#### Evaluation of first Delphi round

Responses were counted and feedback of the participants was evaluated by the core group. Items with < 70% acceptance were excluded from the template or re-evaluated in the core group, especially if items nearly reached the threshold of 70%. Additional comments and suggestions were evaluated, revised and integrated within the relevant domains by the core group. The following guiding principles were taken into account during this process:

I. Improve the wording and language
II. Requests for adding a new item or
III. Requests for deleting an item or aspect of it and
IV. Requests for merging different items or aspects. The expert group ensured that any modification did not result in the omission of an objective that was considered relevant by the majority of the Delphi panel.

#### Second Delphi round

The second Delphi round was conducted in March 2019. Participants received an update of the first Delphi round, which consists of 19 items (data not shown). The same procedure of rating and analysis was used as in the first Delphi round.

## Results

Altogether, 25 raters, consisting of 20 experts invited to participate in the Delphi process, and 5 experts of the core group, confirmed their willingness to participate in the Delphi survey which was conducted in 2 rounds. In the first round, the experts nominally rated 29 items attributed to 4 domains and 13 sub-domains whether they are regarded important enough to be included in a European Collaborative Interprofessional Framework or not.

The agreed recommendations for a collaborative interprofessional capability framework for prevention and management of frailty are summarized in Table 2. It contains 25 items structured in 4 domains and 13 sub-domains of capabilities.

**Table 2:** Collaborative Interprofessional Capability Framework for Prevention and Management of Frailty developed by the Joint Action ADVANTAGE and the European Geriatric Medicine Society (EuGMS)

*Final framework*
*1. Understand frailty*
*1.1. Definition and prevalence*
1.1.1. Knowing that frailty is an age-associated condition of reduced resilience and increased vulnerability to adverse events
1.1.2. Knowing that frailty can be defined through the “frailty phenotype” and the “cumulative deficit” models of frailty
*1.2. Disability, multimorbidity and dependency*
1.2.1. Understand the concept of frailty as a multidimensional condition and recognize its individual nature and stages, including all determinants of health identified by the WHO (CSDH, 2008)
1.2.2. Understand that pre-frailty and frailty are potentially reversible with recognized transitional stages from robust through dependency/disability to the end of life
1.2.3. Knowing that the trajectories of frailty are influenced by lifestyle and other factors, with geriatric syndromes such as confusion, falls, incontinence, impaired mobility and polypharmacy having a complex multidirectional relationship with frailty
*1.3. Personal impact*
1.3.1. Understanding the multidimensional, heterogeneous nature of frailty and its complex multidirectional relationship with many different aspects of a person’s life (including multimorbidity, functional ability, physical health, psychosocial health and cognitive function)
*2. Identification of frailty*
*2.1. Screening, diagnosing and assessment*
2.1.1. Apply common instruments, including those suggested in the Frailty Prevention Approach (FPA) document, to support the identification and assessment (CGA) of frailty as part of an integrated care approach to managing frailty
2.1.2. Knowing that the assessment of frailty should include the consideration of the potential use of assistive technology (AT)
2.1.3. Understand the importance of early recognition and timely management of frailty and its associated signs and symptoms
*3. Person*-*centred collaborative working*
*3.1. Person*-*centred approaches including communication*
3.1.1. Understand that person-centred care includes all elements of a person’s life that are important to them and enables shared decisions in consideration of persons’ priorities
3.1.2. Demonstrate effective communication with older people, family and carers to achieve shared decision-making and to support carers in their individual care-giving role.
*3.2. Collaborative and integrated working*
3.2.1. Be able to share information with other professionals, including an older person’s wishes, in a timely and appropriate manner, considering issues of capacity, consent and confidentiality
3.2.2. Be able to work in partnership with others towards a common goal, exploring and integrating the views across multidisciplinary teams and organizations to deliver care in a coordinated and integrated way, showing an understanding of the role of others
*4. Managing frailty and its prevention*
*4.1. Preventing and reducing the risk of frailty progression*
4.1.1. Know evidence-based interventions to improve independence and quality of life for people at risk of or living with frailty
4.1.2. Be able to measure, monitor and report important measures of frailty outcomes in different settings including all determinants of health
*4.2. Living well*
4.2.1. Understand the concept and principles of a community development, asset-based approach to care and support for older people at risk of frailty or those already living with frailty
*4.3. Promoting independence*
4.3.1. Be able to provide specific advice and guidance on changing or adapting the physical and social environment to promote independence and ensure physical safety, comfort and emotional security
*4.4. Community skills*
4.4.1. Be able to promote the benefits of developing social skills and engaging with the local community, amongst colleagues and senior managers/board members in relation to improving outcomes for people living with frailty and those important to them
*4.5. Care and support planning*
4.5.1. Understand the importance of care and support planning being a “holistic” and person-centred process at all levels of care that needs to be reviewed regularly
*4.6. Research and evidence*-*based practice*
4.6.1. Understand the reasons for conducting service evaluation and research on frailty and frailty prevention and be able to participate in service evaluation and research in the workplace
4.6.2. Understand how local and national policy and the outcomes of research in frailty care and support can inform and impact on workplace practices and care delivery
*4.7. Leadership in transforming services*
4.7.1. Understand the importance of continuing professional development to ensure the methods used for preventing and managing frailty are robust, valid and reliable
4.7.2. Understand that everyone has a part to play in supporting people living with frailty to have the best possible quality of life
4.7.3. Be able to use people’s feedback and person-centred outcomes to advocate and coproduce investment in services for older people at risk or living with frailty and those supporting them
4.7.4. Recognize the importance of effective clinical governance which involves all stakeholders for overall management of frailty
Reference:
CSDH (2008). Closing the gap in a generation: health equity through action on the social determinants of health. Final Report of the Commission on Social Determinants of Health. Geneva, World Health Organization. Available at: https://apps.who.int/iris/bitstream/handle/10665/43943/9789241563703_eng.pdf;jsessionid=3A37DBC5EE56DD9D1B7AAF33DF8AAAF0?sequence=1 [Last access: April 24th, 2019]

Table 2 shows the final consensus achieved among experts on core capabilities to be addressed to tackle prevention and management of frailty on a multiprofessional level

Domain 1 (six items), covers the understanding of frailty. All items achieved a consensus level greater than 70% in round one. All items (six items) rephrased in round one, achieved full consensus in round two (range between 84 and 100%) without further comments.

Domain 2 (three items) covers the knowledge and skills for screening, assessment and early diagnosis of frailty. One item was excluded at round one due to low consensus (52%). Three items were rephrased, and reached full consensus in the second round (84–100% consensus).

Domain 3 (four items) covers non-technical skills for person-centred care and collaborative working in multi-professional teams. Two items were excluded at round one due to low consensus (68% each). All items (four items) rephrased in round one, achieved full consensus in round two (88–100%) without further comments.

Domain 4 (12 items) covers the knowledge and skills to be achieved by all professionals for taking preventive actions on micro–, meso and macrolevel to prevent and tackle frailty. Interestingly, items included in domain 4 raised the highest level of discussion and, therefore, need for change during rounds one and two: one item did not reach level of significance in round one and was excluded. Six items of round one had to be rephrased. Six items had been suggested by participants for direct inclusion during round one without any rephrasing necessary. Suggestions for those items included in the second Delphi round were raised by many participants simultaneously. The core group revised the suggested phrases. All items reached full consensus in round two, with 84–96% agreement.

## Discussion

There is a clear commitment by the “Science Advice for Policy by European Academies” (SAPEA) for European-wide changes in health and social care delivery based on integrated care throughout the whole life span to effectively impact on healthy lifetime [24]. Similar recommendations have been given by WHO in 2015 where “Integration” addresses longitudinal care pathways for citizens themselves as well as horizontal integration of care interventions through linkage of processes currently delivered in a fragmented way in many health care systems and health care delivery is oriented towards individualized and person-centred treatment goals [25]. This approach implies a strong alignment of professionals integrated in the care of patients at any age, particularly of importance for patients with complex care needs, such as old and vulnerable groups.

The ADVANTAGE JA aims at building a shared understanding among policy makers and stakeholders to develop a common European approach to frailty prevention. Task 8.1 (WP8) was asked to critically appraise the current evidence in the field of education/training for health professionals in the prevention of frailty across the European member states. The consortium showed the absence of evidence for the benefit of educational interventions of staff involved into the care process [3].

Based on this previous work [4, 5], it was assumed by members of the consortium of the ADVANTAGE JA that shared values, knowledge and skills would also serve the goals outlined in the SAPEA report and would actively support the quality of care for people with complex care needs across Europe. It was, therefore, the aim of the group to make use of the broad spectrum of best practice models detected throughout Europe during the work of the Joint Action and to validate content from training programs in the best practice settings and build evidence for a multi-professional European Capability Framework for the care of older citizens using a consensus approach (Table 1). This framework should then serve as common key element for further implementation of management recommendations delivered by the JA and to facilitate the translation of results delivered by the ADVANTAGE JA in EU member states. This approach has also been previously supported by the World Health Organization (WHO) [26].

Table 2 highlights that the final agreed capability framework for frailty prevention and management includes four different domains. Besides a common understanding of frailty as a concept, skills to identify frailty were included in the recommendation (Domains 1 and 2 of the framework, Table 2). The majority of capabilities, however, address person-centred, collaborative and integrated working as communication skills, leadership qualities and awareness for innovation and community development are all non-technical skills. This result of this Pan-European consensus process is aligned with strong representation of non-technical skills needed for interprofessional collaborative practice [5]. Equipping professionals with skills for goal-oriented and smooth communication pathways and adapting flat hierarchies within teams and inbetween teams around older people will build social and human bridges, supporting integrated care.

A main link for the collaborative practice in care for older people is “screening” as well as “comprehensive geriatric assessment” (CGA). Those instruments are major backbones for integration of functionality into standard medical care for older people with complex care needs [27]. Especially CGA has been proven effective for health outcomes and functionality in groups of older people in hospital setting [28]. By nature, geriatricians coordinate teams around older people and see CGA as the gold standard of their clinical management. Furthermore, geriatric medicine nowadays has proven the concept of CGA as core element of evidence for integrated complex care management of older patients [29]. By implementing CGA in different care settings for older people within the public health system, it will be possible to align integrated clinical care as well as corresponding research settings [30].

The capability framework presented and developed under the auspices of the Joint Action ADVANTAGE and EuGMS will allow definition of the specific roles of professionals involved into the process of CGA for different settings. Furthermore, this role clarification will allow standardization procedures for all care processes and give insight into efficacy and effectiveness of integrated care of older people in different settings. In a systematic review currently submitted for publication, the authors showed that there is evidence for efficacy of multi-professional team care when including doctors, nurses and physiotherapists in care teams for older people [31]. This makes the current work outstanding as only few publications in the literature currently address the effect of inclusion of different professions in the care teams, such as dieticians, social workers and others. However, many domains included in the CGA touch upon expertise of those professions not initially included in a multi-professional team and only little information is available on role modelling and responsibilities within the multi-professional care teams.

Research shows interprofessional collaboration improves patient outcomes, patient safety, and staff morale while decreasing hospital admissions, length of hospital stays, and staff turnover [32]. Most probably, this is one of the reasons why the current European Health Programme [33] includes a strong focus on integrated care, aiming to improve patient experience and outcomes of care and effectiveness of health systems. Within this concept, it is expected that involved team members must collaborate effectively to achieve sustainability of cross-sectoral complex care interventions. In this context, the framework presented here is pioneering work. It includes shared knowledge, skills and attitudes for many professions involved in the integrated care pathway for many older citizens.

The Joint Action ADVANTAGE provides a European guide on how to preserve capacity in ageing societies on a public health level. However, the multi-dimensional nature of frailty and functional decline raises the need for a holistic and multi-dimensional approach and increases the need for involvement of different stakeholders in distinct care settings. Basic knowledge but also the capability to work in synchrony with frail older peoples’ and their families’ values and goals are necessary [34]. The work presented provides the framework in which all professions around older people should be trained and may, therefore, serve as hallmark for translation of the results of the JA ADVANTAGE in many health and social care systems across Europe. The work presented here is aligned with recommendations on evidence-based management for integrated care for older people (ICOPE) in community to avoid loss of intrinsic capacity, recently launched by WHO [35]. The translation of the recommendations from ICOPE guidelines as well as the capability framework, presented in this publication, into curricula of different professions will be the next step to foster integration of the capabilities into daily practice. Ideally, interprofessional education is used for future training [36].

Developing the health and social care workforce needed for future generations of European citizens is demanding. It is important to see health workforce planning as a process that engages the main stakeholders in assessing needs for change and in devising strategies to achieve those changes. Addressing and focusing on regional and national needs implies more than producing more workers; scaling up can be achieved by improving competences, changing skills mix and by augmenting productivity. For sustainability of these developments, it will be necessary to evaluate the intervention set and to see health work force development as part of quality assurance in health care systems.

The main strength of this study is the attainment of a consensus from a broad spectrum of European stakeholders, ranging from political representatives, to experts in the field of ageing and health, academia as well as practitioners of different settings and care systems across European countries. Working in harmony between professions towards commonly shared therapeutic goals and adapting therapeutic targets in an integrated way throughout lifespan of older citizens will help to personalize care as recommended by many official bodies. The applicability of this agreed framework outside the EU is currently unclear, hypothesis generating and may represent a potential limitation of this study.

## Conclusion

The study describes one of the first, if not “the first” agreed Pan-European multi-professional capability framework for frailty prevention and management developed and supported by the JA ADVANTAGE and the European Geriatric Medicine Society (EuGMS). This framework potentially offers the possibility to many European stakeholders involved in the care process of older citizens on all public health levels to integrate the capabilities outlined into curricula and foster integrated care delivery for older people across Europe.

The framework has a strong focus on person-centred, collaborative and integrated working as communication skills, leadership qualities and awareness for innovation and community development are all non- technical skills. Implementing these capabilities in curricula will be the next step. Working together in daily clinical practice but also on a transnational level and tailoring educational programs for many professions involved into older care will be the focus of the work for the incoming decade.
